# Operational disruption in healthcare associated with software functionality issue due to software security patching: a case report

**DOI:** 10.3389/fdgth.2024.1367431

**Published:** 2024-03-14

**Authors:** Md Shafiqur Rahman Jabin

**Affiliations:** ^1^ Department of Medicine & Optometry, Linnaeus University, Kalmar, Sweden; ^2^ Faculty of Health Studies, University of Bradford, Bradford, United Kingdom

**Keywords:** patient safety, healthcare quality improvement, software issue, training, system integration, system design, software update, workflow disruption

## Abstract

Despite many benefits, the extensive deployment of Health Information Technology (HIT) systems by healthcare organizations has encountered many challenges, particularly in the field of telemetry concerning patient monitoring and its operational workflow. These challenges can add more layers of complexity when an unplanned software security patching is performed, affecting patient monitoring and causing disruption in daily clinical operations. This study is a reflection on what happened associated with software security patching and why it happened through the lens of an incident report to develop potential preventive and corrective strategies using qualitative analyses—inductive and deductive approaches. There is a need for such analyses to identify the underlying mechanism behind such issues since very limited research has been conducted on the study of software patching. The incident was classified as a “software functionality” issue, and the consequence was an “incident with a noticeable consequence but no patient harm”, and the contributing factor was a software update, i.e., software security patching. This report describes how insufficient planning of software patching, lack of training for healthcare professionals, contingency planning on unplanned system disruption, and HIT system configuration can compromise healthcare quality and cause risks to patient safety. We propose 15 preventive and corrective strategies grouped under four key areas based on the system approach and social-technical aspects of the patching process. The key areas are (i) preparing, developing, and deploying patches; (ii) training the frontline operators; (iii) ensuring contingency planning; and (iv) establishing configuration and communication between systems. These strategies are expected to minimize the risk of HIT-related incidents, enhance software security patch management in healthcare organizations, and improve patient safety. However, further discussion should be continued about general HIT problems connected to software security patching.

## 1 Introduction

“A software patch or fix is a quick-repair job for a piece of programming designed to resolve functionality issues, improve security, or add new features” (1). Software patching is a growing key aspect of today's computing envi­ronment (2), particularly in the healthcare environment (3) in which the volume, complexity, and number of configurations have increased consid­erably. A number of challenges associated with software security patching have been encountered in modern healthcare, including delayed patch applications (4), vulnerability scanning, assessment, and prioritization (5). The consequences of such problems due to software patching are enormous, such as causing delays in healthcare management and even risks to patient safety (4, 6). However, the underlying mechanism behind these issues is still unknown in most cases; for example, why and how delays occur while applying those patches (4). In addition, very limited research has been conducted on the study of software security patching, particularly in the context of healthcare.

HIT systems were deployed with the vision of making care delivery safer and more efficient by reducing adverse events and improving accuracy (7). The HITs have improved several dimensions of healthcare quality, such as enhancing the security and confidentiality of personal health information (8), improving patient safety, and increasing efficiency and effectiveness (9). Despite their numerous benefits, the introduction of HITs has encountered substantial problems, including planning, design, implementation, and management (10, 11).

Several studies of implementation science have indicated that the deployment of HIT systems might be successful in one setting but not in others (12). New and often unexpected problems arise, compromising the quality of healthcare and requiring diligent attention and awareness whenever a new technology or solution is introduced (13). Several pieces of evidence suggest that different HIT systems, such as radiology information systems (10, 11, 14) and e-prescribing systems (15), can pose serious consequences, ranging from workflow interruptions (16) and patient inconvenience (9) to multiple patient harm (17). Similarly, patient monitoring systems, such as an ECG monitoring system, can encounter various challenges, including system integration, complex computational needs, and patient/user resistance (18). While some studies suggested that the accuracy and reliability of remote patient monitoring systems can be questioned (19), others reported that security and privacy could also be the major challenges of these systems (20).

HIT systems, particularly those used in patient monitoring, such as central monitoring systems and alarm detectors, are commonly endorsed as the solution to many of the problems encountered by the Emergency Department or intensive care unit (21). Healthcare professionals, such as nurses, heavily depend on such systems, which allow them to monitor the vital signs of multiple patients on the same screen without being physically present in the patient room (22). While there is some evidence for the clinical benefits of this efficient system, there is reported evidence that patient monitoring systems can cause various challenges to patients, compromising healthcare quality (23).

The incident reporting process ensures reflection on what has happened, why it has happened, and how it might have been minimized (13). Sometimes, the incidents can act as an “early warning system” by identifying new issues before reaching the patient (24). They can be used as a basis for devising preventive and corrective strategies and strategies to prevent them from harming the patients (13). Incident reporting may also play a key role in improving the patient safety culture of a healthcare organization with the local follow-up of incidents (25). This necessitates qualitative analysis of the free-text narratives or anecdotes using inductive and deductive techniques. The inductive approach may include content analysis, whereas the deductive method may comprise the classification of the critical aspects of the qualitative data by feeding them into an existing framework, such as the HIT Classification System (HIT-CS) (26).

Since little research has been performed on software security patch management, there is an urgent need for qualitative analysis to explore the issue. Therefore, this case report will present how inappropriate planning of software patching can affect the patient monitoring system and cause disruption in day-to-day clinical operations and care delivery through the lens of an incident report. The report will also provide some useful insights for practitioners and researchers to understand what and where strategies are necessary to better support the patch management process.

## 2 Methods

### 2.1 Data collection

The incident (presented in Box 1) was reported in an electronic incident management database for medical devices, i.e., the reidarMTP. The reidarMTP aims to make essential information on medical devices readily available for the healthcare environment, primarily in Sweden and the Nordic countries, such as Denmark, Norway, Finland, and Iceland. The reidarMTP is operated by a voluntary association of Clinical Engineering departments in Swedish hospitals and is handled by certified staff trained to report such information into an open database. The information in the web database is anonymous and freely available to all healthcare professionals for quality improvement, education, and training (27, 28).

Box 1 **This software security patching-related incident was reported to the reidarMTP by an anonymous user showing responses to the following categories of information.**

**Description of the incident**

The program X, which is used, among other things, for security patching of patient monitoring systems, does not work well, which means problems when patching is to be done. Patching of Windows is done by Medical Technology to maintain high IT security.
During patching, restarts often occur, and during these restarts, no central patient monitoring can take place. This means that patching is carefully planned together with the business in order to disrupt as little as possible. These operational disturbances mean risks for telemetry patients in particular. Central monitoring and alarm detectors do not work during the patching, so the departments need to set aside extra resources to compensate for this.
X's job in patching is to schedule and initiate patching. The program indicates the client/monitoring centre patching status in green or red. During the last patching, you got a green light, meaning that the patching is complete and there is no pending restart. It was then assumed that the work was finished. The following morning, however, about half of the centres/clients handled the day before were red. This means that a further restart is needed, which is a major operational disruption for the departments that were unable to plan for this.
A major flaw in X is that Medical Technology cannot see when the patches are applied, how long this will take, how many patches will be applied, and whether they are applied at the same time or not. This makes it very difficult to make an assessment of how much operational disruption a patching will entail.

**Summary of cause investigation**

Patching of PC clients is done every six months, an interval deemed appropriate by Medical Technology. If patching was performed more often, not as many/large patches would have to be applied on each occasion, which could reduce the risk of what happened in this case. However, this would mean that the operations are affected by and need to plan for operational disruption more often.
Medical Technology's assessment is that patching more often overall would mean a greater impact on the operations.

The incidents are generally categorized into several different fields, entailing different sets of information. The first category includes the date, day, and time of events, an incident description with a short subject line, for example, “patching software for patient monitoring does not work well.” The second category is about the type of products involved in the incident, such as product name, manufacturer, software version, serial/batch number, etc. The third category comprises investigation, such as a summary of cause investigation, a summary of actions, and a summary of follow-up. The final and fourth categories consist of classification or risk assessment, including risk of medical damage and underlying cause.

The incident has been filtered and illustrated in Box 1 in two fields: “incident description,” which was reported by anonymous healthcare staff, and “summary of cause investigation,” i.e., an internal investigated narrative of the reported incident. The report was delivered in Swedish and translated into English by a linguistic expert who is proficient in both Swedish and English. The technical nature of the content through the translation process was taken into consideration with the help of consensus by the linguistic expert and the principal investigator. To maintain anonymity, the name of the software product has been masked by “X”.

### 2.2 Data analysis

The incident was analyzed using both deductive and inductive approaches. The deductive approach included an existing framework proposed by Magrabi et al., i.e., the HIT-CS (26). The HIT-CS has particularly been tailored to address the issues arising from HIT in healthcare for deconstructing incidents, classifying HIT-related issues, and extracting meaningful information. Issues can be classified based on human or technical-related problems, whereas technical challenges can be grouped into hardware and software-related problems (26). The HIT-CS was used to identify the type of software issue, the type of consequence, and the contributing (human) factor. The inductive approach involved content analysis. The application of the existing framework, i.e., HIT-CS and content extraction analyses, were managed on a semantic level—the exact content of the incident was taken into consideration, and no assumptions were made about the latent underpinnings of the incident report. Both of these approaches helped develop a set of preventive and corrective strategies that could potentially minimize future occurrences of these risks.

## 3 Results

The HIT-CS was used for incident classification to enhance transparency and understanding. The incident was classified as a technical issue, i.e., “software functionality”, and the consequence of the incident was categorized as “incident with noticeable consequence but no patient harm”, and the contributing factor was “integration with clinical workflow”.

Using the content analysis, the contributing factors, mitigating factors, and patient/ organizational outcomes were identified. The contributing factor was identified to be the software update, i.e., software security patching. Although the incident did not cause any harm to patients directly, the operational disruption was clearly indicated in the incident description. To mitigate such software issues, one has to be mindful not to use many or large patches if security patching is to be done frequently, i.e., every six months. Another mitigating factor was identified, i.e., a contingency plan for frequent operation disruption. There was no patient outcome described in the narrative; however, the organizational outcome was determined to be severe disruptions in the clinical workflow for several weeks.

## 4 Discussion

The issue of software patching and the coordination of different components have become a common phenomenon in modern healthcare systems. The outcomes of these issues can cause workflow disruptions or delays in healthcare delivery and serious risks to patient safety (4, 6). For example, an empirical investigation in the healthcare sector indicated delays in applying software security patches, particularly in the patch deployment phase, due to coordination delays relating to technology, people, and organization (4). Another study proposed a similar theory, i.e., a lack of in-depth understanding of socio-technical aspects of the patching process and patching decisions causing delays in applying security patches (29). To mitigate such delays and maintain a timely security patch management process, the studies recommended coordination and interdependent software/ hardware components and the decisions made by multiple stakeholders involved. The delays can also be minimized by designing and developing computer-aided supportive tools (4, 29).

Jabin et al. demonstrated in 2019 that HIT incidents occurred at each step of the medical imaging workflow process and that human and technical factors play a role in problems related to patient details (16). Such disruptions in the workflow process cause significant delays in patient treatments, patient inconvenience, and risks to patient safety, including repeat images resulting in unnecessary radiation and even additional workload for radiographers, i.e., repeat reconstruction of radiographic images. A recent study indicated that approximately 41% of the total sample of incidents had a staff/organization-related outcome with a clear indication that workflow disruptions resulted in additional system/service/resource use and delays in using facilities/service/systems (7). These delays in treatment or procedure further cause delayed diagnosis, treatment initiation, impact, and monitoring. Such delays can even cause delays in the decision-making process regarding further treatment options—continuation, discontinuation, or change in treatment. This means that once an incorrect shred of information or document is initiated into the HIT system, an “automation bias” tends to be considered correct (30).

A robust mechanism of system resilience and high-reliability organizations must address system flaws or software-related issues, including software patches, in a timely manner (31). Ensuring a robust mechanism means that similar types of errors are not repeated in the future, which will further guarantee that preventive and corrective interventions are applied at a system level (32). Therefore, such a system-wide approach and reliable systems can quickly identify and fix the issues related to HIT systems, minimize the stress and dissatisfaction of healthcare professionals, and thus improve healthcare quality (33).

### 4.1 Implications for practice

Based on the system approach and through the lens of social-technical aspects of the patching process associated with HIT systems, such as the patient monitoring system, we propose the following 15 preventive and corrective strategies, which are grouped under four key areas (as outlined in Box 2). The key areas are (i) preparing, developing, and deploying patches; (ii) training the frontline operators; (iii) ensuring contingency planning; and (iv) establishing configuration and communication between systems.

Box 2 **Preventive and corrective strategies to mitigate and manage the risk of HIT incidents.**

***Preparing, developing, and deploying patches***

▪ Understand the problem beyond what the reporter outlined and identify the source of the vulnerability before developing a patch.
▪ Work with the original developer of the system/component to ensure designing/creating the right fix.
▪ Carefully plan for a stable fix with full attention to security and without the loss of any functionality
▪ Create a deployable and installable (by end-user) package using automated patch management solutions and ensure the patches do not conflict with the previous patches in the same system/component.
▪ Establish a wide distribution of the patch quickly and efficiently to end users once the deployable package has been verified to fix the problem and all regression and compatibility testing has been secured.
▪ Track the status of the patch download and installation by the service/management tool to help determine if the patch is successfully installed or if it is initiating any compatibility issues with other applications.

***Training the frontline operators***

▪ Set up training for healthcare professionals prior to deploying any patches as part of preparing for situational awareness
▪ Provide professionals with training updates as part of professional development following software patches

***Ensuring contingency planning***

▪ Carefully plan any system changes to mitigate disruption to the regular workload and ensure contingency planning
▪ Ensure appropriate IT support and access to appointed IT experts in a timely manner in case of any unexpected failure within the facility
▪ Establish comprehensive plans and emergency operation modes for managing any new and unforeseen downtimes
▪ Set up a robust mechanism to communicate planned or unplanned power failure to all healthcare professionals involved in the service
▪ Ensure safety standards and sufficient escalation procedures to deal with the issues that cause patient harm

***Establishing configuration and communication between systems***

▪ Configure HIT systems (central monitoring system and alarm detectors) to ensure they are interoperable and communicate with each other.
▪ Ensure access to the care plan/history/details of telemetry patients at the time of operational disruption

(i) Preparing, developing, and deploying patches—software security patch management in large and complex systems like healthcare is a challenging process that engages numerous stakeholders and involves multiple interdependent socio-technical decisions. A number of steps need to be followed systematically (as outlined in Box 2) in order to overcome patching security vulnerabilities. A systematic review of software security patch management based on 72 included studies identified 14 socio-technical challenges and 18 solution approaches, tools, and practices mapped into the process of software security patch management (5). The study drew some conclusions on various opportunities for practitioners to adopt new solutions to overcome common challenges and understand the variations of common practices.

**Figure 1:**
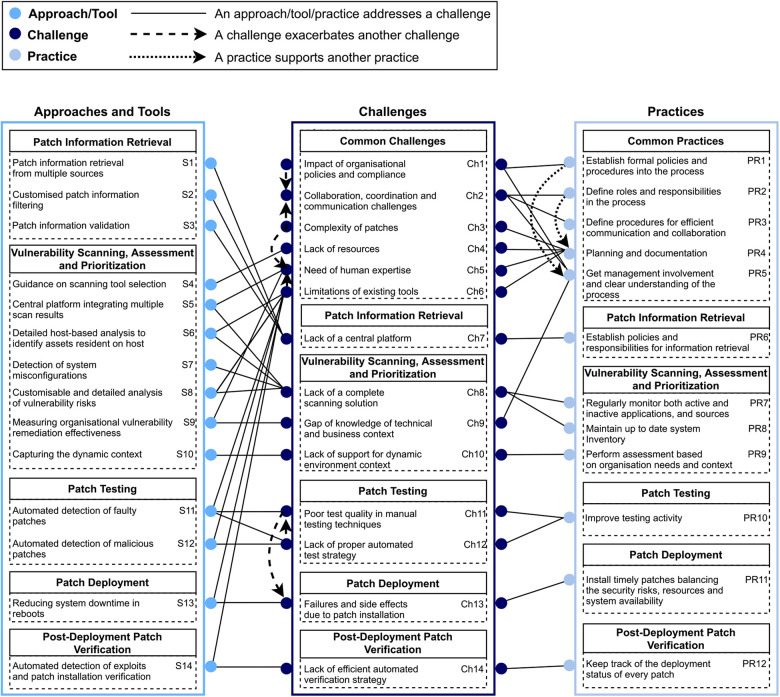
A mapping of challenges onto solutions. Reprinted with permission from Dissanayake et al. (34), licensed under CC BY 4.0, https://doi.org/10.48550/arXiv.2012.00544.

We recommend that the readers use this systematic review as a guide or handbook for software security patch management—preparing, developing, and deploying patches*.* Figure 1 presents a mapping of the findings to enable the readers to identify the relationships between challenges and proposed solutions (5).

(ii) Training the frontline operators—providing training and education to healthcare professionals, ideally in cooperation with HIT vendors, prior to deploying any patches will mitigate the risk of patient harm.

Several studies suggest that training healthcare staff should be included in the planning process to effectively respond to a disruption. For example, a study on HIT risk and resilience states that “an organization's ability to effectively respond to a disruption not only depends on how effective it was in the planning process, but also how effective it was with its preparation, trials, and the training of their staff, which is often neglected” (35). Another study by Jabin et al. in 2019 identified the need to set up a process for initial and ongoing training of the operators to minimize the risks associated with human factors-related errors and workflow interruptions (13).

(iii) Ensuring contingency planning—a greater focus on stakeholder engagement in all aspects of healthcare practice, such as care providers, practice, quality, and/or departmental managers; accreditors, IT staff, and professional associations who set the standards of practice should be in place. This should include appropriate backup and emergency plans/measures to minimize disruption to regular care delivery, communicate unplanned power failure, and manage unexpected downtimes (33).

For example, a survey of US-based healthcare institutions focused on sharing HIT-related best practices and shared insight about Electronic Health Record (EHR)-related downtimes (36). The survey found that the majority of organizations experienced extended EHR-related downtimes, and most institutions implemented partial comprehensive contingency plans to mitigate the risks of unexpected EHR downtimes. The study concluded that “contingency planning” should be a routine part of all EHR-enabled healthcare organizations; we should eventually prepare for continuity of operations and ensure safe and effective healthcare.

(iv) Establishing configuration and communication between systems—configuration between different HIT systems, such as central monitoring systems and alarm detectors, should be considered at the time of design and purchase of systems (33).

Several other studies recommended to establish configuration and communication between systems as one of the strategies to overcome HIT-related issues and ensure safe and effective healthcare. For example, a study on e-prescribing-related challenges suggested ensuring software quality in an interfaced, networked healthcare environment since Lack of communication and appropriate configuration between systems was identified to be the major problem (15). Another similar study proposed that appropriate HIT configuration must be established to ensure access to prior studies, data integrity, and appropriate interfaces for record migration (17).

These strategies will be beneficial in improving healthcare quality and mitigating the risk of patient harm from issues with the HIT systems, such as the telemetry patient monitoring system. This recommendation guide will help set aside additional resources to compensate for any major operational disruptions; thus, the need for such a guide for healthcare professionals is urgent.

### 4.2 Strengths and limitations of the study

The major strength of this study is the use of both qualitative approaches, deductive (existing framework) and inductive (content analysis), permitting the investigator to obtain more detailed information from the incident report. Both of these approaches are most suitable due to their salient features for the qualitative data, i.e., free text narratives (17, 31); therefore, no other approach, such as Machine learning, could be applied. Although Machine learning could potentially extend the principle of qualitative analysis, offering a promising technique to scale up the coding process, one has to keep in mind that the study was on a single incident (not a set of incident reports) (37). Moreover, the application of both these approaches helps to minimize the potential subjective bias in devising 15 preventive and corrective strategies.

The incident report considered for this study was voluntary with its inherent limitations, including subjective bias, reporters’ lack of knowledge of the HIT systems/ software security patching, or inclination to provide a comprehensive report. In addition, a follow-up communication to glean additional information could not be conducted due to the anonymity of the reporter. Notwithstanding these limitations, the findings and the devised strategies can be considered as alerts to enlighten healthcare digitalization in Sweden to adopt the culture of digital safety and effectiveness. This also implies that the lessons learned from this case report can be useful and pertinent to adopt elsewhere for overall healthcare quality improvement and patient safety (7, 17, 32).

## 5 Conclusion

Major operational disruptions in the clinical workflow for several weeks may take place as a result of insufficient planning and complex processes (many/large/frequent patches) of software security patching. Such workflow interruptions occur due to inadequate training for frontline operators for unexpected system failure, lack of foresight, and poor understanding of HIT system integration into practice. To mitigate the identified risk, the software security patch management must be aligned with the context of clinical workflow. The first step of this alignment requires a proper understanding and consideration of proper planning, preparing, developing, and deploying patches. This should be followed by setting up the training process for healthcare professionals prior to any software patches and ensuring contingency planning to cope with any unexpected failures. The strategies should also include the configuration of HIT systems to ensure they are interoperable and communicate with each other.

As a multitude of settings, i.e., technology, people, and healthcare organizations, are potentially affected, it is challenging to specify in further detail. However, further discussion should be continued, emphasizing the need for adaptability in technology and healthcare practices and general HIT problems connected to software security patching. There is also a need to reinforce the necessity of systematic incident reporting as a fundamental practice for improving healthcare quality and patient safety.

## Data Availability

The incident data has already been presented in Box 1. However, the data presented in this study are available on request from the corresponding author.
